# High‐Performance Aluminum Fuels Induced by Monolayer Self‐Assembly of Nano‐Sized Energetic Fluoride Vesicles on the Surface

**DOI:** 10.1002/advs.202401564

**Published:** 2024-05-05

**Authors:** Ruibin Wang, Lichen Zhang, Xiaodong Li, Lixiang Zhu, Zilong Xiang, Jin Xu, Dichang Xue, Zitong Deng, Xing Su, Meishuai Zou

**Affiliations:** ^1^ School of Materials Science and Engineering Beijing Institute of Technology No. 5 South Zhongguancun Street, Haidian Beijing 100081 China

**Keywords:** energetic fluoride vesicle, high‐performance aluminum fuels, long‐range force, monolayer self‐assembly, surface nanostructure

## Abstract

Surface modification is frequently used to solve the problems of low combustion properties and agglomeration for aluminum‐based fuels. However, due to the intrinsic incompatibility between the aluminum powder and the organic modifiers, the surface coating is usually uneven and disordered, which significantly deteriorates the uniformity and performances of the Al‐based fuels. Herein, a new approach of monolayer nano‐vesicular self‐assembly is proposed to prepare high‐performance Al fuels. Triblock copolymer G‐F‐G is produced by glycidyl azide polymer (GAP) and 2,2′‐(2,2,3,3,4,5,5‐Octafluorohexane‐1,6‐diyl) bis (oxirane) (fluoride) ring‐open addition reaction. By utilizing G‐F‐G vesicular self‐assembly in a special solvent, the nano‐sized vesicles are firmly adhered to the surface of Al powder through the long‐range attraction between the fluorine segments and Al. Meanwhile, the electrostatic repulsion between vesicles ensures an extremely thin coating thickness (≈15 nm), maintaining the monolayer coating structure. Nice ignition, combustion, anti‐agglomeration, and water‐proof properties of Al@G‐F‐G(DMF) are achieved, which are superior among the existing Al‐based fuels. The derived Al‐based fuel has excellent comprehensive properties, which can not only inspire the development of new‐generation energetic materials but also provide facile but exquisite strategies for exquisite surface nanostructure construction via ordered self‐assembly for many other applications.

## Graphical abstract

GAP‐Fluoride‐GAP molecules are self‐assembled into nano‐vesicles in specific solvents, in which they are stably dispersed due to the electrostatic repulsion of the vesicle surface. The long‐range attraction between Aluminum and the fluorine segments makes the vesicles firmly adhere to the aluminum surface. This ensures a monolayer coating of the nano‐vesicles. Such surface nanostructure with high surface area can greatly improve the combustion performance and hydrophobicity of aluminum powder.

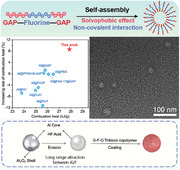

## 1 Introduction

The pursuit of superior performance has always been the focus in the field of energetic materials.^[^ ^1^ ^]^ With the development of material science, higher expectations of novel energetic materials have been raised in the applications of aerospace, civil blasting, and national defense.^[^ ^2^ ^]^ Because of its low toxicity, high abundance, and high energy density,^[^ ^3^ ^]^ Al often serves as the metallic fuel in solid propellants, which determines the key properties such as burning rate and specific impulse.^[^ ^4^ ^]^ However, due to the high chemical reactivity of Al powder, the formation of the outer alumina (Al_2_O_3_) layer is usually inevitable during preparation, transportation, and application.^[^ ^2a^ ^]^ The melting point for outer Al_2_O_3_ (2072 °C) is much higher than that of Al (660 °C).^[^ ^5^ ^]^ This causes significantly enhanced ignition temperature. Besides, the Al core is inevitably expanded and ruptured during combustion,^[^ ^2b^ ^]^ causing large‐size agglomeration and thus drastically reduced combustion efficiency. The deposition and agglomeration of Al powder induced by incomplete combustion can also bring safety hazards for engines.^[^ ^6^ ^]^ Therefore, how to reduce the adverse effects of alumina shell on the Al‐based fuels is the core technology in the field of energetic materials, which has great research significance.

In order to resolve the above issues, surface modification by perfluoride seems to be an efficient way.^[^ ^7^ ^]^ The high electron affinity to Al^[^ ^7^, ^8^ ^]^ and the pre‐ignition reaction (PIR)^[^ ^7^, ^9^ ^]^ make it possible for perfluorides to effectively destroy the alumina shell and inhibit the agglomeration of combustion residues.^[^ ^7b^ ^]^ Considering the non‐energetic nature of perfluorides,^[^ ^7c^ ^]^ GAP containing energetic azide group (─N_3_) is often concerned with further enhancing the energetic level of Al‐base fuels.^[^ ^10^ ^]^ Multiple methods for synthesizing energetic perfluoride coating on the surface of Al have been developed including physical blending,^[^ ^7d^ ^]^ etherification,^[^ ^11^ ^]^ esterification,^[^ ^12^ ^]^ epoxy addition,^[^ ^7d^ ^]^ and etc. Resultantly, the combination of perfluoride and GAP has the potential of synchronously improving multiple properties of Al‐based fuels in terms of ignition, combustion, and energy release.

However, thresholds remain for the current surface modification strategies of Al‐based fuels. There is a lack of exquisite design and systematic study on the energetic perfluoride coating microstructure. The thickness of the coating layer on Al‐based fuels is large, which is essentially greater than 100 nm. More importantly, the microstructure of facial coating is highly non‐uniform and non‐adjustable in terms of surface topography. These usually reduce the performance stability, combustion heat, and effective loading capacity of Al fuels, which significantly limit the applicable properties of solid propellants. Therefore, without being limited to traditional energetic fluorine chemistry, it is crucial to conduct research on special surface morphology design and regulation for Al‐based fuels.

Accordingly, we proposed a novel surface modification strategy for Al fuels via of the incorporation of energetic fluoride vesicles. In this work, the triblock G‐F‐G polymer was adopted. It tended to form self‐assembled vesicles in a certain liquid environment. The repulsive electrostatic forces among the vesicles,^[^ ^13^ ^]^ as well as long‐range attractive forces^[^ ^14^ ^]^ between vesicles and Al powder, allowed vesicles to be uniformly distributed on the surface of Al powder to form an monolayer, high‐specific area (increased by 66.3%), ultra‐thin (≈15 nm), uniform (thickness deviation <5 nm) coating layer. The effective Al content in the coated Al‐based fuel could surpass 97%, which was higher than the existing surface modified Al‐based fuels. Compared with raw Al powder, the combustion heat of the coated Al‐based fuel could be increased by 9.5%, and the combustion efficiency was increased to 91.1% (increased by 8.4%). This work offers a new structural design strategy for surface modified Al‐based fuels with improved overall performance, which opens new scopes for the design of advanced energetic materials in the future.

## 2 Results and Discussion

### 2.1 Synthesis and Characterizations of the G‐F‐G Triblock Copolymer

**Figure 1:**
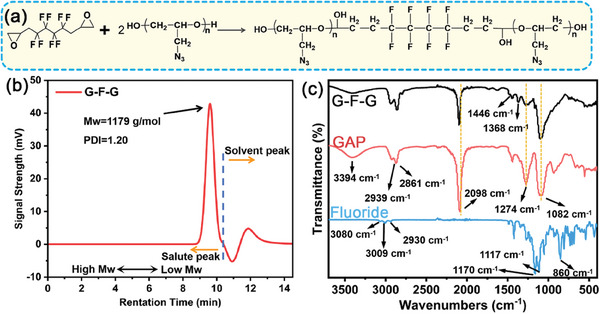
The schematic diagram of ring‐opening addition reaction of a) G‐F‐G triblock copolymer; b) GPC curve of G‐F‐G; c) FTIR curves of G‐F‐G, GAP, and fluoride.

In this work, GAP and 2,2′‐(2,2,3,3,4,4,5,5‐Octafluorohexane‐1,6‐diyl) bis (oxirane) were used to synthesize G‐F‐G triblock copolymer, as shown in **Figure** **1**a. The two end groups of GAP were hydroxyl groups, while the two end groups of fluoride were epoxy groups. Epoxy groups could react with hydroxyl groups via ring‐open addition chemistry. According to the characteristic molecular weight and functional groups of the synthetic product, the formation of G‐F‐G could be proved by GPC (Figure 1b), FTIR (Figure 1c), and F‐NMR (Figure S1, Supporting Information). The detailed results from FTIR spectra are shown in Table S1 (Supporting Information). The FTIR spectra of fluoride and GAP proved the existence of hydroxyl, azide, methylene skeleton, and ether bonds, which confirmed the formation of chemically bonded fluoride/GAP compound. After the ring‐open addition of GAP and fluoride, G‐F‐G triblock copolymer was formed. In Figure S1 (Supporting Information), a comparison was made by F‐NMR of fluoride and a G‐F‐G copolymer. It was observed that the chemical environment of F remained unchanged with two distinct types. This implied that C‐F did not underwent any chemical reaction. As for the GPC data of G‐F‐G (Figure 1b), the weight average molecular weight was 1179 g mol^−1^, which was close to the theoretical molecular weight value of G‐F‐G (1139 g mol^−1^). The polydispersity index (PDI) of G‐F‐G was 1.20, which was due to the polydispersed GAP (Figure S2, Supporting Information). It was clear that the fluoride/GAP feeding ratio was highly similar to the fluoride/GAP chemical composition (1:2 in molar ratio) in the product. This was due to the high reactivity and product stability of ring‐opening addition chemistry.^[^ ^15^ ^]^ Though the ring‐open addition reaction gave rise to the newly formed hydroxyl side groups, our experimental results showed that the products still tended to form the linear G‐F‐G triblock copolymer structure, rather than branched compounds.

The conjugated effect of azide groups activated the hydroxyl groups, making them highly reactive to the epoxy groups of the fluoride and facilitating the formation of block copolymer. The newly generated side hydroxyl groups in the middle of G‐F‐G had significantly lower reactivity to the epoxy groups. The reasons were attributed to the aspects as shown below. First, fluorine had a strong electron‐drawing effect.^[^ ^16^ ^]^ Both side hydroxyl groups in G‐F‐G and epoxy groups in fluoride were affected by the adjacent perfluorinated chain segments, reducing the activity for nucleophilic addition reaction. Second, the side hydroxyl groups displayed a certain steric hindrance effect, which further hindered the reaction with fluoride.^[^ ^17^ ^]^ Third, according to molecular collision theory, the molecular weight and chain length of the derived G‐F‐G were much larger than that of GAP, which greatly reduced its effective collisions during the reaction and highly inhibited further chain propagation of G‐F‐G.^[^ ^18^ ^]^ As a result, fluoride tended to react with GAP instead of G‐F‐G product, generating a linear G‐F‐G triblock copolymer structure^.[^ ^19^ ^]^ To further confirm the above statement, we conducted density functional theory (DFT) simulation calculation and the results were shown in simulation calculation part (Section S3, Supporting Information). Compared to the reaction between fluoride and GAP, it was highly difficult for the side reaction between fluoride and G‐F‐G to occur in our work. These results indicated that the G‐F‐G block copolymer was successfully synthesized.

### 2.2 Formation and Characterization of G‐F‐G Triblock Copolymer Vesicles

Biomacromolecules such as proteins contain both hydrophilic and hydrophobic segments, which could induce specific aggregative effects.^[^ ^20^ ^]^ The surface charge gave rise to the long‐range electrostatic repulsion, preventing from overstocking.^[^ ^21^ ^]^ And the specific aggregative effect leads to long‐range Van der Waals forces,^[^ ^22^ ^]^ helping biomacromolecules to adsorb on the surface of bio‐tissues. These unique features address various functions. Inspiringly, we tried to construct nano‐sized G‐F‐G vesicular coating on the surface of Al fuels. It was necessary for the derived G‐F‐G triblock copolymer to be dissolved in a suitable organic solvent for modifying the Al surface. When G‐F‐G was placed in absolute ethanol (AE), it was highly insoluble. G‐F‐G was seemingly dissolved in tetrahydrofuran (THF) after a long period (>4 h). However, precipitates gradually appeared at the bottom of the container after staying still for a day, indicating poor solubility. When DMF was used to dissolve G‐F‐G, the solution seemed to be uniform and stable, and there was no precipitate after several days.

**Figure 2:**
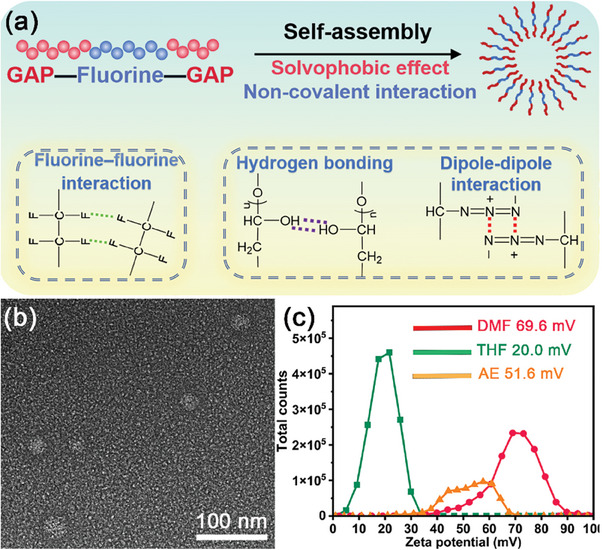
Schematic illustration of a) vesicular self‐assembly; b) Cryo‐TEM image of vesicles self‐assembled by G‐F‐G triblock copolymer; c) The zeta potential of G‐F‐G in DMF, THF, or AE with corresponding average values.

It was worth noting that, linear G‐F‐G molecules tended to self‐assembly and form vesicles in N,N‐dimethylformide (DMF). As for the fluorine segments (solvophobic) in the middle,^[^ ^23^ ^]^ the intermolecular interactions were dominated by the fluorine‐fluorine interactions.^[^ ^19^, ^24^ ^]^ As for the GAP segments (solvophilic) at both ends, the dipole‐dipole interactions among azide groups and the hydrogen bonds among hydroxyl groups dominated.^[^ ^25^ ^]^ Because the average polymerization degree (n) of polydisperse GAP segments in this work was quite small (n ≈ 4.67), even minor change in n could bring in big influence on its chain length and the asymmetry of G‐F‐G. In the vesicles, the relatively long GAP segments tended to head outward while the relatively short ones tended to head inward.^[^ ^26^ ^]^ There was spacing inside the vesicles, which were filled with DMF solvent. Therefore, G‐F‐G could assemble into a unique vesicular structure with the solvophobic intermediate membrane and solvophilic inner/outer coronas,^[^ ^27^ ^]^ as the schematic illustration shown in **Figure** **2**a. In order to investigate the morphology of G‐F‐G vesicles in DMF, Cryo‐TEM was conducted. As shown in Figure 2b, the G‐F‐G vesicular spheres with the diameter ≈27 nm dispersed in the DMF solvent could be clearly observed. Typical core‐shell structure for vesicles could be detected by specifically treated G‐F‐G dispersion via Cryo‐TEM (Figure S3, Supporting Information). The statistical average particle size of 31.7 nm (solvation diameter) was also obtained by DLS as seen from Figure S4 (Supporting Information), which was close to the results told by Cryo‐TEM.

It could be seen from Figure 2c that the vesicles in DMF possessed the highest electro‐positivity (69.6 mV) among the tested materials. Large absolute values of zeta potential often indicated strong electrostatic repulsion and thus the vesicles could remain stable in DMF solvent.^[^ ^28^ ^]^ Conversely, the vesicles would essentially tend to agglomerate in THF. Unlike AE and THF, DMF was a good solvent for GAP marginal segments but a bad solvent for fluorine segments in the middle.^[^ ^23^ ^]^ Due to the strong electron‐drawing effect of the gathering fluorine‐containing segments,^[^ ^29^ ^]^ the GAP ends tended to show electro‐positivity. According to the DLVO theory, the vesicle–vesicle interactions included electrostatic repulsion forces and Van der Waals forces.^[^ ^30^ ^]^ High surface charge density (zeta potential) was indicative of strong electrostatic repulsion forces among the approaching vesicles.^[^ ^30^ ^]^ Sufficient electrostatic repulsion forces were able to overcome the Van der Waals forces, which helped to maintain the stable dispersion of vesicles in DMF.^[^ ^30^, ^31^ ^]^ These zeta potential results indicated that the formation of vesicles was highly solvent‐selective. The solubility parameters between the solute and the solvent, as well as their polarity, played important roles in the formation of vesicles. The estimation of solubility parameter for G‐F‐G via the group contribution method was conducted and the details can be seen in Table S2 (Supporting Information). The results indicated that G‐F‐G preferred to form vesicles in highly polar DMF solvent.

### 2.3 Formation and Characterization of G‐F‐G Vesicle‐Coated Al Power

**Figure 3:**
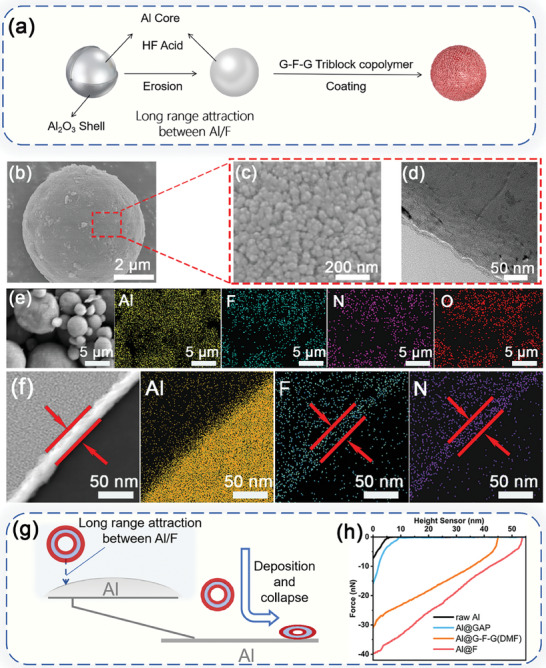
The schematic illustration of the synthetic process of a) Al@G‐F‐G; The SEM image of an b) Al@G‐F‐G(DMF) particle c) with a magnified surface and d) cross‐sectional morphology by TEM; e) EDS images of Al@G‐F‐G(DMF); f) Cross‐sectional EDS images of Al@G‐F‐G(DMF); g) The depiction of the adsorption mechanisms between G‐F‐G vesicles and Al; h) The quantitative nanomechanical mapping curves of long‐range attraction forces.

The Al powder was treated by HF for removing the facial alumina layer. And an unique solvent‐assisted vesicular self‐assembly strategy was adopted for the surface modification of Al by G‐F‐G copolymer (**Figure** **3**a). The successful modification of Al with G‐F‐G was confirmed by SEM (Figure 3b). Comparing to the other control samples (Figure S5, Supporting Information), tightly arranged coating structure could be merely observed for Al@G‐F‐G(DMF) in Figure 3c. To demonstrate the thickness of coating layer on Al@G‐F‐G(DMF), four Al@G‐F‐G(DMF) particles were randomly selected and cut for observing the cross‐sectional areas (Figure 3d; Figure S6, Supporting Information). The coating thickness was ≈15 nm, smaller than 27 nm from Cryo‐TEM. Comparing Figure 3c,d, the transverse size of the dried vesicles deposited on the surface of Al varied from 18 to 26 nm, which was larger than the coating thickness. This indicated that the G‐F‐G vesicles were successfully deposited on the Al surface. The vesicles were dried and the corresponding vesicular structure was collapsed by losing the solvent in the core.^[^ ^32^ ^]^ It was additional evidence for the formation of G‐F‐G vesicles in DMF. As for EDTA titration (Table S3, Supporting Information), the weight content of G‐F‐G coating on Al@G‐F‐G(DMF) was calculated to be merely 2.27%. These indicated the formation of monolayer vesicular coating, which could be reflected by other means of characterization. FTIR was carried out for Al@G‐F‐G(DMF) (Figure S7, Supporting Information). The characteristic peaks at 2098 cm^−1^ for the ─N_3_ groups in G‐F‐G were clearly shown, which could prove the existence of facial modification. As shown in Figure 3e, the F, N, and O elements in the ultra‐thin vesicular coating were evenly distributed on the surface of Al. As for the EDS mapping on the cross‐sectional area for Al@G‐F‐G(DMF) (Figure 3f), F and N elements densely appeared at the thin coating layer on the surface of Al. The fine spectum of Al, F, N, C, and the total XPS spectra of Al@G‐F‐G(DMF) are shown in Figure S8 (Supporting Information), treated by curve fitting. Al 2p (Figure S8b, Supporting Information) had two chemical states corresponding to Al (71.6 eV) and Al_2_O_3_ (74.7 eV), respectively.^[^ ^33^ ^]^ The characteristic peak of C 1s (Figure S8c) appeared at 284.6, 286.2, and 288.8 eV, which were attributed to the ether bonds in G─F─G, C─H/C─C and C─F_2_. There was one characteristic peak for N 1s at 400.5 eV, correspondng to C─N_3_ (Figure S8d, Supporting Information). The characteristic peak of F 1s appeared at 687.4 eV, which attributed to C─F (Figure S8e, Supporting Information). Furthermore, Figure S9 shows the XRD patterns of Al@G‐F‐G(DMF), except the five peaks characteristic for Al (38.4°, 44.6°, 65.1°, 78.2°, and 82.5°), there were two peaks (13.9°, 31.7°) corresponding to AlF_1.5_(OH)_1.5_(H_2_O)_0.375_ due to HF erosion.^[^ ^7^, ^34^ ^]^ Such unique nanoscale facial microstructure on Al gave rise to fairly high specific surface area of 9.23 m^2^ g^−1^ for Al@G‐F‐G(DMF), which was increased by 66.3% compared to that of raw Al (Table S4, Supporting Information). These phenomena were drastically different from the other control samples (Figure S5) and the reported coated Al fuels, which often had thick and consecutive facial coating structure.^[^ ^7^, ^34^, ^35^ ^]^ The coating thickness for Al@G‐F‐G(AE), Al@G‐F‐G(THF) and Al/F/GAP reached 100, 270, and 277 nm (Figure S10, Supporting Information), respectively. According to EDTA titration, the weight content of organic coating on Al@G‐F‐G(AE), Al@G‐F‐G(THF) and Al/F/GAP were 5.84%, 5.32%, and 13.7%, respectively. Without the formation of vesicles, these control samples had much thicker and heavier coating than Al@G‐F‐G(DMF).

The formation of such unique monolayer vesicular coating were attributed to two aspects. On the one hand, the electrostatic repulsion as discussed before effectively prevented them from overstocking even with increasing G‐F‐G concentration (0.75 mg mL^−1^, more details can be seen in Figure S11, Supporting Information).^[^ ^36^ ^]^ On the other hand, strong long‐range attraction between G‐F‐G vesicles and Al played an important role in the formation of the coating layer. As shown in Figure 3g, though the outer GAP layer directly contacted the Al surface, the attraction was dominated by the non‐attachable electron affinity between Al and the fluorine segments^[^ ^7d^ ^]^ in the middle of G‐F‐G vesicles.

The origin of long‐range attraction was described as follows. The perfluorinated chain segments in the middle of G‐F‐G molecules gathered to form the intemedium layer with amplified electron‐withdrawing effect.^[^ ^37^ ^]^ Therefore, the G‐F‐G copolymer molecules were highly polarized, perfluorinated intermediate membrane was electronegative.^[^ ^7d^ ^]^ The synergy of electronegativity and vesiculation effect had the capability of creating patch‐charge surface heterogeneities^[^ ^38^ ^]^ at the organic/inorganic interfaces. This highly facilitated the formation of long‐range attractive forces once the G‐F‐G vesicles came into contact with the Al surface.^[^ ^39^ ^]^ The long‐range attraction between G‐F‐G vesicles and Al was evidenced by atomic force microscopy (Figure 3h).^[^ ^40^ ^]^ The quantitative nanomechanical mapping of raw Al, Al@GAP, Al@F and Al@G‐F‐G(DMF) were carried out. It was found that the attractive force of raw Al to the probe was merely 7 nN and the effective action distance was 5 nm. Similarly, GAP showed faint attraction and short effective action distance to the probe. Distinctively, the attraction force induced by fluoride to the probe and the effective action distance were significantly enhanced up to 40 nN and 53 nm, respectively. When it came to Al@G‐F‐G(DMF), despite the outer layer of GAP segements with faint attraction, the corresponding attractive force (31 nN) and effective action distance (45 nm) were significantly higher than those of raw Al. Clearly, even being blocked by the non‐attractive segments, long‐range attraction allowed the formation of monolayer G‐F‐G vesicular coating on Al with firm attachment.

### 2.4 Thermal Reaction Properties

**Figure 4:**
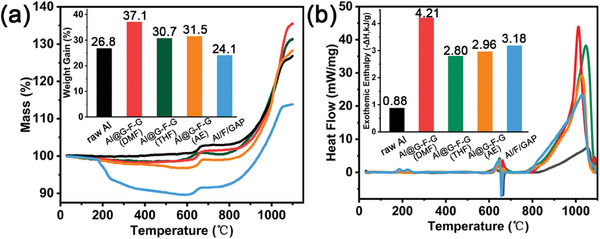
a) TGA curves and comparison of weight gains, b) DSC curves and exothermic enthalpy energy levels in 660–1100 °C of raw Al, Al@G‐F‐G(DMF), Al@G‐F‐G(THF), Al@G‐F‐G(AE), Al/F/GAP with the heating rate of 10 °C min^−1^ in the air atmosphere.

The thermal reaction properties of Al, Al@G‐F‐G(DMF), Al@G‐F‐G(THF), Al@G‐F‐G(AE), Al/F/GAP at room temperature (RT) to 1100 °C were measured by simultaneous thermal analyzer, as shown in **Figure** **4**. Whether it was raw Al powder or coated Al powder, there was no significant weight change from RT to 150 °C, indicating thermal stability within such a temperature range. The TG curve of all samples could be divided into four stages. As for RT ≈ 600 °C, the oxidation process at this stage was not violent. Slow oxidation dominated and the weight gain was quite small. Different from the raw Al, the weight of the coated Al‐based fuels was decreased at 170–600 °C, which was attributed to the thermal decomposition of organic coating. The weight loss of Al/F/GAP at this stage was over 10 wt.% while that of Al@G‐F‐G(DMF) was 1.56 wt.%. As for 600–660 °C, the temperature surpassed the melting point of raw Al, and the solid‐to‐liquid phase transition occurred for Al. At ≈660 °C, all tested samples showed a sign of weight gain, indicating that the oxidation reaction on the surface of Al was activated. When the temperature exceeded 800 °C, due to the complete decomposition of the surface coating, the oxidation reaction of Al led to a rapid increase in weight and intense heat release.^[^ ^7^, ^34^ ^]^ The mass of Al, Al@G‐F‐G(DMF), Al@G‐F‐G(THF), Al@G‐F‐G(AE), and Al/F/GAP were increased by 26.8%, 37.1%, 30.7%, 31.5%, and 24.1%, respectively, during the weight gain stage at 660–1100 °C. The weight gain rate of Al@G‐F‐G(DMF) was increased by 37.1% due to the G‐F‐G vesicular coating layer, which was attributed to two aspects. On the one hand, the G‐F‐G coating layer was fairly thin and its content was fairly low comparing to the other control samples. This significantly improved the loading content of Al, which thereby enhanced the weight gain of Al@G‐F‐G(DMF). On the other hand, the specific surface area of Al@G‐F‐G(DMF) sample was significantly increased due to the granulate morphology of vesicles, which helped to increase the contact area between Al powder and air. Therefore, the oxidation of Al was highly facilitated at air atmosphere.

It could be seen from the heat release curves from DSC tests that the melting heat absorption peaks of Al powder samples all appeared near the melting point of 660 °C, and their heat release enthalpy at 660–1100 °C were 0.88 kJ g^−1^ for raw Al, 4.21 kJ g^−1^ for Al@G‐F‐G(DMF), 2.80 kJ g^−1^ for Al@G‐F‐G(THF), 2.96 kJ g^−1^ for Al@G‐F‐G(AE), and 3.18 kJ g^−1^ for Al/F/GAP, respectively. The organic coating tended to increase the heat release enthalpy and the maximum heat flow could exceed 40 W g^−1^. The coating layer of Al/F/GAP was the thickest (Figure S10, Supporting Information) and heaviest (Table S3, Supporting Information) among the tested samples and its exothermic enthalpy was higher than those of Al@G‐F‐G(AE) and Al@G‐F‐G(THF). It indicated that as for the traditional modification methods, it was necessary to increase the content of energetic coating for improving exothermic enthalpy.^[^ ^41^ ^]^ Distinctively, Al@G‐F‐G(DMF), with the lowest coating mass, displayed higher exothermic enthalpy than that of Al/F/GAP, indicating the advantages of the self‐assembled vesicles on Al in terms of thermal properties. In connection with the Al powder coating structure mentioned before, the monolayer vesicular structure on the surface of Al@G‐F‐G(DMF) led to stable oxidation and efficient heat release during the heating process. In brief, Al@G‐F‐G(DMF) samples exhibited exceptionally high weight gain rate and heat release enthalpy at the expense of minimal coating mass.

### 2.5 The Ignition and Combustion Performance

**Figure 5:**
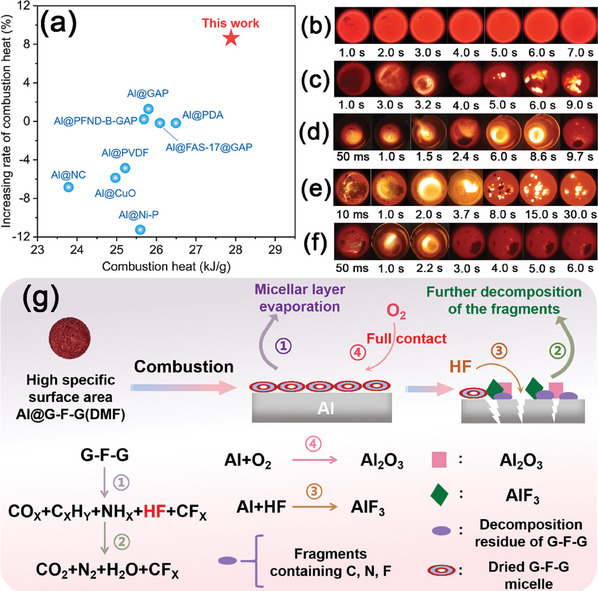
The advantages of this work in comparison with some representative studies a) including Al@PVDF, Al@CuO, and Al@PDA corresponding to ref. [42], Al@NC and Al@GAP corresponding to ref. [43], Al@Ni‐P corresponding to ref. [44], Al@PFND‐B‐GAP corresponding to ref. [34] and Al@FAS‐17@GAP corresponding to ref. [11]; b) Snapshots of ignition testing process for raw Al, c) Al@G‐F‐G(THF), d) Al@G‐F‐G(AE), e) Al@G‐F‐G(DMF), and f) Al/F/GAP in a high‐temperature melting furnace; g) The schematic illustration of the detailed combustion mechanisms for Al@G‐F‐G(DMF).

The results of combustion heat and ignition tests are shown in **Table** **1**. The theoretical combustion heat of raw Al was 30.96 kJ g^−1^. The combustion heat of energetic GAP in this work was evaluated as 21.60 kJ g^−1^. The weight content of the coating layer in each sample could be obtained by EDTA titration (Table S3). The statistical data of combustion heat values could be referred to Figure S12 (Supporting Information). The combustion efficiency of each sample could be calculated according to the derived data. The actual combustion heat of raw Al, Al@G‐F‐G(THF), Al@G‐F‐G(AE), Al@G‐F‐G(DMF), and Al/F/GAP were 25.61, 24.35, 27.08, 28.05, and 24.10 kJ g^−1^, respectively. The corresponding combustion efficiencies of Al were 82.7%, 79.2%, 88.5%, 91.1%, and 81.7%, respectively. Al@G‐F‐G(DMF) displayed the best combustion heat and combustion efficiency among the tested samples. Compared with raw Al, the combustion heat of Al@G‐F‐G(DMF) coated with vesicles were significantly increased by 9.5%. Besides, the combustion heat and combustion efficiency of Al@G‐F‐G(DMF) were significantly increased comparing to those of Al@G‐F‐G(THF) by 15.2% and 15.0%, respectively. According to TEM images (Figure S10, Supporting Information) and TG curves (Figure 4), it indicated that inferior combustion performance of Al@G‐F‐G(THF) was attributed to poor and unstable surface coating structure. Clearly, with minimal coating thickness and mass, Al@G‐F‐G(DMF) with orderly arranged G‐F‐G vesicles on Al surface was likely to effectively improve the combustion heat and combustion efficiency. The promoting effect of the monolayer vesicular coating on the combustion heat of Al‐based fuels was outstanding among the current works (**Figure** **5**a).

**Table 1:** The data of combustion heat and ignition test.

Sample	Oxygen bomb calorimeter test		Ignition test		
	Heat release [kJ g^−1^]	Combustion efficiency [%]	Minimum ignition temperature [°C]	tI*) [s]	tB*) [s]
Al	25.61	82.7%	—	—	—
Al@G‐F‐G(THF)	24.35	79.2%	750	1.6	6.4
Al@G‐F‐G(AE)	27.08	88.5%	760	0.05	9.7
**Al@G‐F‐G(DMF)**	**28.05**	**91.1%**	**710**	**0.01**	**>20**
Al/F/GAP	24.10	81.7%	720	0.05	3.0

tI* ignition delay time at the minimum ignition temperature; tB* burning duration at the minimum ignition temperature; — was not ignited until 1000 °C.

Figure 5b–f shows the snapshots of the ignition tests for Al, Al@G‐F‐G(THF), Al@G‐F‐G(AE), Al@G‐F‐G(DMF), and Al/F/GAP taken by a high‐speed camera. During tests, the raw Al was merely melton, but not ignited even at the temperature of 1000 °C in the high‐temperature melting furnace (Figure 5b). Distinctively, the minimum ignition temperature of the surface modifieid Al powder samples dropped below 800 °C. Among them, the vesicular self‐assembly modified Al@G‐F‐G(DMF) covered by orderly self‐assembled mecelles displayed the lowest ignition temperature (710 °C), the shortest ignition delay time (10 ms) and the longest combustion time (>30 s). The flame area of Al@G‐F‐G(DMF) was the largest among the tested samples. Once Al@G‐F‐G(DMF) got in touch with the bottom of the furnace, a large number of sparks suddenly bursted out, which accompanied with gas waves. After 1.0 s, massive dazzling flame overflow occurred, which lasted till 3.7 s. Subsequently, the combustion of residual Al@G‐F‐G(DMF) carried on with many bright points of flame, which could last for 30.0 s. As for the control samples, the ignition delay time of Al@G‐F‐G(THF) was greatly extended to 1.6 s with small flame area and the intermittent combustion process (Figure 5c). The ignition delay time of Al@G‐F‐G(AE) was 50 ms (Figure 5d). The corresponding flame was greatly weakened after 2.4 s. Though it was reignited at 6.0 s, it was then completely extinguished after 9.7 s. The above results indicated that the high surface area of the orderly self‐assemblied vesicles on the Al surface played an vital role in the combustion of Al@G‐F‐G(DMF). It allowed Al@G‐F‐G(DMF) to react violently and produce a large amount of gas as soon as it contacted the bottom of the furnace. Similar to fireworks, Al@G‐F‐G(DMF) not only burned vigorously at the beginning (Figure 5e), but also continued to be oxidized after the flame has spilled out. The ignition delay time of Al/F/GAP via physical blending method was 50 ms, but the combustion was fairly weak with small flame area and faint light (Figure 5f). The flame was completely extinguished after 3.0 s. In contrast, the coating layers on the control samples were thick and uneven, leading to intermittent and incomplete combustion.

In order to study the corresponding reaction mechanisms, the TG/FTIR curves of Al@G‐F‐G(DMF) (Figure S13, Supporting Information) were analyzed and the detailed combustion mechanisms were depicted by Figure 5g. The region of 4000–3500 cm^−1^ corresponded to the absorption band of water vapor.^[^ ^34^ ^]^ The peaks of 2850–2990 cm^−1^ belonged to hydrocarbons. Bands at 2360 and 2300 cm^−1^ were attributed to the stretching vibrations of CO_2_ and CO.^[^ ^45^ ^]^ Bands at 1160 and 665 cm^−1^ were attributed to C─F and N─H bonds,^[^ ^46^ ^]^ respectively.^[^ ^47^ ^]^ The peaks at 1750, 1510, and 1243 cm^−1^ were attributed to the stretching vibrations of C═O, C═C, and C─O bonds.^[^ ^45^ ^]^ Negative peaks appeared due to the generation of water vapor in the environment.^[^ ^7d^ ^]^ At ≈200 °C, the weight loss rate reached the maximum. Afterward, the peaks for hydrocarbons became obvious, which were due to the generation of hydrocarbons upon decomposition. After 400 °C, the weight loss ceased and the peaks for hydrocarbons almost disappeared from the FTIR curves. The peak intensity variation of CO_2_ and CO at 200, 400, and 660 °C indicated that CO_2_ and CO were produced along with the release of hydrocarbons. So, the thermal decomposition of G‐F‐G coating was the main cause of weight loss at this stage. Around the melting point of Al at 660 °C, the weight of the sample began to go up with a sharp peak ≈1400–1870 cm^−1^ in the FTIR spectrum. This indicated that the coating layer began to participate in the oxidation of Al. Above 900 °C, the intensity of the peaks for CO_2_ and CO rose rapidly and the characteristic peak for N─H appeared. Fragments containing C and N with aromatic ring structures were likely to appear after the G‐F‐G backbone was destroyed.^[^ ^47^, ^48^ ^]^ These fragments tended to adhere to the surface of molten Al. With increasing temperature, they were further decomposed into CO_2_ and N─H compounds accompanied by certain heat release. This corresponded to the exothermic peak on the DSC curve (Figure 4). Such exothermal behavior tended to accelerate the thermal oxidation of molten Al powder by O_2_. Therefore, the weight gain rate ≈900–1000 °C for was significantly higher than that of raw Al. Accordingly, the monolayer and vesicular G‐F‐G coating structure on Al@G‐F‐G(DMF) had a significant promoting effect on the combustion process. On the one hand, the monolayer structure could avoid facial overstocking of organics, which helped to effectively activate the ignition and combustion of Al.^[^ ^30^ ^]^ On the other hand, the vesicular structure gave rise to fairly high specific surface area (Figure S4, Supporting Information). This could highly improve the surface chemical reactivity of GAP segments, leading to an in‐advance exothermic reaction.^[^ ^49^ ^]^ And this could highly enhance the facial adsorption capacity of air, greatly increasing the solid‐gas contact area and accelerating the combustion reactions.^[^ ^50^ ^]^

**Figure 6:**
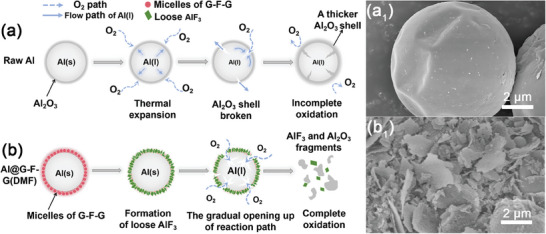
Schematic illustration of the formation and morphology for the combustion debris corresponding to a) raw Al and b) Al@G‐F‐G(DMF).

**Figure** **6** illustrates the shell‐breaking mechanisms during the combustion process of vesicles‐coated Al@G‐F‐G(DMF) compared with raw Al. Figure 6a shows that the dense alumina on the surface of Al hindered the oxidation of Al. As the temperature rose, though the liquid Al inside could help to break the alumina shell, the alumina layer was continuously regenerated and further oxidation was hindered. Therefore, the untreated Al powder underwent weak oxidation and alumina shell deformation, leaving a large amount of unoxidized Al. Figure 6b illustrates the promoting effect of orderly self‐assembled G‐F‐G vesicles on Al combustion. The vesicular coating layer made the surface of Al@G‐F‐G(DMF) more active.^[^ ^50^ ^]^ The thermal decomposition of vesicles gave rise to a tightly coated AlF_3_ layer in the form of loose sheets as shown in Figure S14 (Supporting Information). XRD curves corresponding to the combustion product of Al@G‐F‐G(DMF) in Figure S9 (Supporting Information) also confirmed the formation of the AlF_3_ phase. On the surface of Al@G‐F‐G(DMF), fluoride temporarily existed as AlF_1.5_(OH)_1.5_(H_2_O)_0.375_, which was finally converted to AlF_3_ upon combustion. This loose AlF_3_ layer facilitated the subsequent contact of Al powder with oxygen and promoted oxidation. It was worth noting that a characteristic peak for γ‐Al_2_O_3_ appeared in the combustion product of Al@G‐F‐G(DMF). γ‐Al_2_O_3_ possessed a fairly loose microstructure compared to other form of alumina. Such microstructure of γ‐Al_2_O_3_ on the surface of Al@G‐F‐G(DMF) also contributed to the effective adsorption of oxygen and high reactivity during combustion.^[^ ^51^ ^]^

The above‐mentioned mechanisms helped to constantly clear the combustion pathway during combustion. Therefore, unlike raw Al, the combustion product of Al@G‐F‐G(DMF) was fully shattered and the spherical shape of the Al powder was completely destroyed as shown in Figure 6b. Figure S15 (Supporting Information) shows the particle size distribution of the combustion debris, which reflected the agglomeration state of each sample after combustion. Although the raw Al was not ignited, the melton Al could still break through the Al_2_O_3_ shell, causing severe agglomeraion. The size of derived aggregates originating from raw Al was within the range of 200–1000 µm. In contrast, Al@G‐F‐G(DMF) showed a nice anti‐agglomeration tendency. The corresponding size of the combustion debris was below 20 µm. A large proportion (20–30%) of debris displayed a particle size below 3 µm, which was even smaller than the initial particle size of raw Al. Compared with other modified samples, it was obvious that the surface vesicular structure contributed to the superior anti‐agglomeration property of Al‐based fuels. The size distribution for the combustion product of Al/F/GAP, Al@G‐F‐G(AE), and Al@G‐F‐G(THF) were similar. It indicated that the effect of anti‐agglomeration was not solely determined by the content of the F element in the coating layer. Because even if the coating mass was merely 2.27 wt.%, Al@G‐F‐G(DMF) also displayed overwhelming combustion properties due to its unique vesicular coating structure.

### 2.6 Hydrophobicity

**Figure 7:**
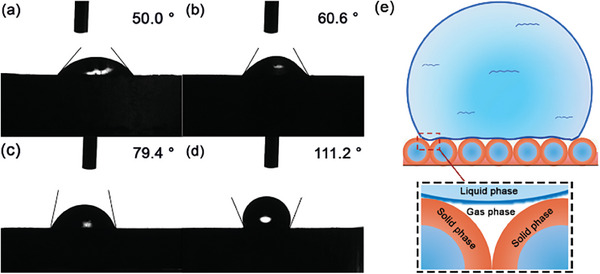
Illustration of contact angles of a) raw Al, b) Al@GAP, c) Al@F, and d) Al@G‐F‐G(DMF) to water; e) Schematic illustration for the hydrophobic mechanisms of Al@G‐F‐G(DMF) (e).

Contact angle tests were carried out for the derived Al‐based fuels and the data are shown in **Figure** **7**. With θ = 90° as the boundary to judge the wettability, when θ > 90°, the solid‐gas surface tension was lower than the liquid–gas surface tension.^[^ ^52^ ^]^ Therefore, the droplets shrank and gathered into beads along the solid surface, which were easy to slide on the solid surface. In this case, the surface was difficult to be wetted, showing hydrophobicity. When θ < 90°, the surface was prone to wet by water, showing hydrophilicity. As for Figure 7a–d, though the hydrophobicity of the surface of Al powder could be slightly improved by either GAP or fluoride single coating, the corresponding contact angle (θ) was still lower than 90° and the surface of the coated powder could be infiltrated by water. In contrast, the contact angle of Al@G‐F‐G(DMF) with orderly self‐assembled vesicles to water was increased sharply to 111.2°, which was 61.2° higher than that of the raw Al. Similar to lotus leaves, the high hydrophobicity of Al@G‐F‐G(DMF) was attributed to not only the highly hydrophobic fluorine segments, but also the facial mastoid microstructure (Figure 7e). Such unique mastoid microstructure highly facilitated the formation of a three‐phase hybrid nanostructure once contacting water as shown in Figure 7e. The key step for many superhydrophobic surfaces was to construct surfaces (i.e., lotus leaf) with micro and nano‐sized rough structures.^[^ ^53^ ^]^ This nanostructure could greatly reduce the contact area between the liquid phase and the solid phase, increasing the hydrophobicity of the material.^[^ ^54^ ^]^ And the intermediate regions filled with the gas phase further lifted the water droplets with increased distance between the liquid phase and solid phase,^[^ ^55^ ^]^ leading to a “semi‐suspended” state. Therefore, the hydrophobicity of Al@G‐F‐G(DMF) was significantly enhanced compared to that of the other control samples. This could effectively inhibit the water‐induced aging and extend the shelf life in the storage of Al powder, which addressed big significance in the process of transportation and preservation. This also provided important insight for the design of highly hydrophobic coating for Al‐based fuels without compromising the effective Al metal content.

## 3 Conclusion

In this work, a novel Al‐based fuel with facial self‐assembly nanostructure by vesicles was developed. By using a highly polar solvent with a certain solvation effect for asymmetrical triblock G‐F‐G copolymer, nanoscale vesicles were formed via hydrogen bonding, dipole–dipole interaction, and fluorine–fluorine interaction. The self‐assembly and electron‐drawing effect of fluorine led to the vesicle–vesicle electrostatic repulsion and the vesicle‐Al long‐range attraction forces. The vesicles could form a monolayer, orderly arranged, and stable coating on the surface of Al powder. Different from the existing modifying strategies of Al‐based fuels, the thickness and mass fraction of the coating layer could be controlled around the thickness of 15 nm and the content of 2.27 wt.%, respectively. The specific surface area could be significantly increased by 66.3%, which greatly facilitated the ignition and combustion reactions. The combustion heat was enhanced by 9.5% compared to raw Al powder. The minimum ignition temperature was reduced to 710 °C. The ignition delay time was reduced to 10 ms. The combustion duration was greater than 30 s. The surface hydrophobicity of the Al‐based fuel was also significantly improved due to the nanoscale three‐phase structure with vesicular coating. This work not only presented a new strategy to construct a metal fuel with significantly improved overall performance but also provided a new idea for the design of multifunctional self‐assembly interfaces in the future.

## 4 Experimental Section

### Materials

2,2′‐(2,2,3,3,4,4,5,5‐Octafluorohexane‐1,6‐diyl) bis (oxirane) (>98%) was purchased from Tokyo Chemical Industry CO., Ltd (Shanghai, China). Al powder (particle diameter: 3–8 µm), boron fluoride ethyl ether (purity: 46.5% min), and xylene orange (purity: indicator grade) were acquired from Aladdin Chemistry Co., Ltd. (Shanghai, China). GAP (Weight average molecular weight: 480, purity: chemical pure) was purchased from Liming Chemical Engineering Institute Luoyang, China. Dimethyl Sulfoxide (DMSO, GR), tetrahydrofuran (99.5%, super dry, with molecular sieves, H_2_O ≤ 50 ppm), sodium hydroxide (99.0%), ethylenediaminetetraacetic acid (EDTA, 0.5 mol L^−1^, pH 7.4), and ZnCl_2_ (99.95%) were acquired from Meryer Chemical Technology Co., Ltd. (Shanghai, China). Hydrofluoric acid (HF), absolute ethanol (AE), N, N‐Dimethylformamide (DMF) with a purity ≥99.5%, and hydrochloric acid (HCl, 36–38%) were purchased from Sinopharm Chemical Reagent Beijing Co., Ltd. All chemicals were used as received.

### Preparation of G‐F‐G Triblock Copolymer

First, 1.995 g (0.0042 mol) GAP and 20 mL THF were added into a beaker and stirred for 20 min to reach complete dissolution. The solution was poured into a three‐neck flask which filled with dried nitrogen. Then 0.6 mL of boron fluoride solution dissolved in ethyl ether was added into the reaction vessel and stirred for 10 min. Afterward, 443 µL (0.002 mol) 2,2′‐(2,2,3,3,4,4,5,5‐Octafluorohexane‐1,6‐ diyl) bis (oxirane) was dispersed with 30 mL THF and added dropwise at a flow rate of 5 mL h^−1^. The slightly excessive GAP was for the sake of inhibiting potential side reactions. The mixture was under constant stirring at 55 °C for 8 h in an oil bath pan. Then the solvent was evaporated at 70 °C in the oven for 3 h and transferred to the vacuum oven to be dried for 2 hours at 70 °C to obtain the G‐F‐G triblock copolymer. Finally, it was further purified by a chromatographic column.

### Preparation of Al@G‐F‐G

Taking the synthetic process of Al@G‐F‐G(DMF) as an example, 20 mL DMF was poured into a three‐neck flask filled with nitrogen. 2.0 mL diluted hydrofluoric acid (mass fraction of 3%) was added to the reaction vessel and stirred for 10 min until the added substances were evenly mixed. Then 1.0 g Al powder was added into the above solution with the protection of nitrogen gas, facilitating the removal of the facial alumina layer by hydrofluoric acid. Then, 0.15 g G‐F‐G triblock copolymer was dissolved in 20 mL DMF and stirred for 10 minutes to reach fine dispersion. The obtained solution was added to the three‐neck flask dropwise at a flow rate of 20 mL h^−1^ with a constant pressure separator and stirred for 6 h. The product was filtered, washed with anhydrous ethanol, transferred to a vacuum oven at 100 °C for 5 h, and dried to obtain Al@G‐F‐G(DMF). AE and THF were also used as solvents for synthesizing Al@G‐F‐G(AE) and Al@G‐F‐G(THF) samples. The samples solely coated with GAP or fluoride were also synthesized, which were abbreviated as Al@GAP and Al@F, respectively. The sample coated with physically mixed GAP and fluoride (Al/F/GAP) was also synthesized. For each Al‐based fuel sample, the concentration of the organic additives in the DMF solution was kept constant at 3.75 mg mL^−1^. The loading content of organic coating substances (fluoride, GAP, G‐F‐G or GAP/fluoride mixture) was fixed at 15 wt.% with respect to the weight of the raw Al. As for the physically modified sample of Al/F/GAP, the content of GAP and fluoride were set to be 11.3 and 3.7 wt.%, respectively. These were consistent with the chemical composition of fluoride and GAP components in the G‐F‐G copolymer. During solution blending, the stirring rate was set to 400 r min^−1^. The temperature was maintained at 25 °C.

### EDTA Titration

One gram of Al powder sample was accurately weighed and added into a flask, and then 40.0 mL NaOH solution (1.0 mol L^−1^) was added into the flask. The flask was heated and stirred in the oil bath to be completely dissolved. After cooling, HCl solution (1 mol L^−1^) was dropwise added until precipitation occurred. And the addition of HCl solution continued until the precipitation was dissolved again. The derived solution was transferred into a 250 mL volumetric bottle. Deionized water was added until the liquid level reached the scale line.

The above solution (25.0 mL) and 35.0 mL of EDTA solution (0.1 mol L^−1^) were collected and added into a 250 mL conical bottle. Two drops of xylene orange solution (0.2%) were added to the solution so that the solution turned yellow. Afterward, dilute ammonia solution (10 wt.%) was added into the conical bottle until the solution turned purple. HCl solution was subsequently added to adjust the pH and make the solution turn yellow. The derived solution was boiled for a while followed by cooling down to the ambient temperature. The standard solution of zinc salt with the Zn^2+^ concentration of 1 mg L^−1^ was added into the solution drop by drop for titration and evaluation of the excess EDTA. The titration was terminated until the solution was exactly converted from yellow to purple. The titration consumption of the zinc salt standard solution was recorded. The mass fraction of Al could be calculated by the data recorded above.

### Characterization

Scanning electron microscopy (SEM, Hitachi S‐4800, Japan) with energy dispersive X‐ray spectroscopy (EDS) was used to detect the morphologies and elemental composition of Al, Al@G‐F‐G(DMF), Al@G‐F‐G(AE), Al@G‐F‐G(THF), and Al/F/GAP. The surface of all samples was sputtered with Au. The core‐shell structure of the derived Al‐based fuels was analyzed by transmission electron microscope (TEM), as well as the high‐resolution transmission electron microscopy (HRTEM, FEI Tecnai G2 F30, Thermo Fisher Scientific FEI Co., USA) with energy dispersive spectrometer (Oxford XPLORE). The samples were sliced by a focused ion beam (FIB) for HRTEM tests. Cryo‐transmission electron microscopy (Cryo‐TEM) and dynamic light scattering (DLS) were commonly used to show the overall morphology and size contribution of vesicles. To characterize the size distribution of vesicles in diverse solvents including AE, THF, and DMF, the DLS and Zeta potentials were calculated by Malvern Zetasizer Nano ZS ZEN3600. Cryogenic transmission electron microscope (Cryo‐TEM, FEI Talos F200C, Thermo Fisher Scientific FEI Co., USA) was used to intuitively detect the vesicular morphology of G‐F‐G copolymer in solution with the freezing temperature of −80 °C. Apart from G‐F‐G vesicles dispersed in DMF, water was also used to treat vesicle samples for better presenting the core‐shell structure. Typically, 1 mL vesicle dispersion was extracted and added to 3 mL water. After 5 mins’ ultrasonication, the derived sample was then frozen. The obtained water‐treated sample was also evaluated by Cryo‐TEM. Fourier transform infrared (FTIR) tests on 2,2′‐(2,2,3,3,4,4,5,5‐Octafluorohexane‐1,6‐diyl) bis (oxirane), GAP, and G‐F‐G triblock copolymer were conducted with a Nicolet 6700 IR spectrometer. H NMR spectra of 2,2′‐(2,2,3,3,4,4,5,5‐Octafluorohexane‐1,6‐diyl) bis (oxirane), GAP and G‐F‐G triblock copolymer were recorded on a Bruker Avance II 400 MHz instrument using CDCl_3_ as solvent. X‐ray diffraction (XRD) of raw Al, Al@G‐F‐G(DMF), Al/F/GAP, and corresponding combustion products were recorded on a Rigaku MiniFlex 600 and the scanning range was 5°−90°. The binding energy of elements in raw Al and Al@G‐F‐G(DMF) was characterized by X‐ray photoelectron spectroscopy (XPS, PHI QUANTERA‐II SXM, ULVAC‐PHI, Japan). The particle size distributions of Al before and after modification were characterized by a laser particle analyzer (Mastersizer 2000, Malvern). The adhesion forces of Al, Al@GAP, Al@G‐F‐G(DMF), and Al@F were tested by the quantitative nanomechanical mapping function of atomic force microscopy (AFM, Bruker ICON). The tested samples for AFM were pressed into tablets with a pressure of 4 MPa, and the type of probe was SCM‐PIT‐V2 (the one coated with platinum‐iridium alloy). In order to calculate the solubility parameters of G‐F‐G, the density was measured by the AccuPycII1340 automatic gas displacement true densitometer. To study the combustion mechanism of Al@G‐F‐G(DMF), TG/FTIR was measured by NETZSCH STA 449 F5/F3 Jupiter.

Thermogravimetric analysis (TGA) and differential scanning calorimetry (DSC) tests were performed on a simultaneous thermal analyzer (NETZSCH STA 449 F3) to determine the thermal performance of samples under an air atmosphere at a heating rate of 10 °C min^−1^ within the temperature range from 30 °C to 1100 °C. An oxygen bomb calorimeter (Parr 6200) was used to measure the combustion heat of raw Al, Al@G‐F‐G(DMF), Al@G‐F‐G(AE), Al/F/GAP, and Al@G‐F‐G(THF), the combustion heat of each sample was tested five times and the corresponding average values were adopted. In order to simulate the instantaneous high‐temperature reaction of the tested samples in an air atmosphere, 0.3 g samples were added into high‐temperature melting furnace. The minimum ignition temperature, ignition delay time, and combustion duration were obtained by controlling the input temperature. Three parallel experiments were conducted for ensuring reliability. The reaction products of raw Al, Al@G‐F‐G(DMF), Al@G‐F‐G(AE), Al/F/GAP, and Al@G‐F‐G(THF) were collected and characterized by SEM, DLS, and XRD. A contact angle tester (SDC‐350H) was used to evaluate the waterproof properties of the facial coating layers on the Al powders.

## Conflict of Interest

The authors declare no conflict of interest.

## Supporting information

Supporting Information

## Data Availability

The data that support the findings of this study are available from the corresponding author upon reasonable request.
