# Supplementary material for: High‐Performance Aluminum Fuels Induced by Monolayer Self‐Assembly of Nano‐Sized Energetic Fluoride Vesicles on the Surface
Source: Adv Sci (Weinh). 2024 May 5;11(26):2401564. doi: 10.1002/advs.202401564 (PMC11234408; doi:10.1002/advs.202401564)
Supplement: Supplementary file 1 — Supporting Information [file ADVS-11-2401564-s001.pdf]

ADVANCED SCIENCE
Open Access

Supporting Information

for *Adv. Sci.*, DOI 10.1002/advs.202401564

High-Performance Aluminum Fuels Induced by Monolayer Self-Assembly of Nano-Sized Energetic Fluoride Vesicles on the Surface

Ruibin Wang, Lichen Zhang, Xiaodong Li, Lixiang Zhu, Zilong Xiang, Jin Xu, Dichang Xue, Zitong Deng, Xing Su and Meishuai Zou**

Supporting Information

High-performance aluminum fuels induced by monolayer self-assembly of nano-sized energetic fluoride vesicles on the surface

Ruibin Wang, Lichen Zhang, Xiaodong Li, Lixiang Zhu, Zilong Xiang, Jin Xu, Dichang Xue, Zitong Deng, Xing Su*, Meishuai Zou*

School of Materials Science and Engineering, Beijing Institute of Technology, No. 5 South Zhongguancun Street, Haidian District, Beijing, 100081, China

*Corresponding author, e-mail: 6120220003@bit.edu.cn (X. Su), zoums@bit.edu.cn

Corresponding authors.
E-mail addresses: 6120220003@bit.edu.cn (X. Su), zoums@bit.edu.cn (M. Zou).

Table of Contents

1 Supplementary Tables

Table S1 .... S2

Table S2 .... S3

Table S3 .... S3

Table S4 .... S6

2 Supplementary Figures

Figure S1 .... S6

Figure S2 .... S6

Figure S3 .... S7

Figure S4 .... S7

Figure S5 .... S7

Figure S6 .... S8

Figure S7 .... S8

Figure S8 .... S9

Figure S9 .... S10

Figure S10 .... S10

Figure S11 .... S11

Figure S12 .... S11

Figure S13 .... S11

Figure S14 .... S12

Figure S15 .... S12

Figure S16 .... S13

3 Simulation calculation of G-F-G synthetic chemistry S13

1 Supplementary Tables

Table S1. The correspondence between FTIR peaks and chemical groups of G-F-G.

Wavenumber (cm^{-1})	Chemical group	Wavenumber (cm^{-1})	Chemical group
2930[1]	-CH-	1200~1100[2]	symmetric/asymmetric -CF_2- stretching
860[3]	epoxy group	2098[1, 4]	-N_3
1350~1200[5]	stretching vibration of C-F	3394	-OH
900~700[5]	bending vibration of C-F	2939/2861[6]	symmetric/ asymmetric -CH_2- stretching vibrations
1082/1274[1, 7]	stretching vibration peaks of -O-	933	bending vibration of -O-

In the FTIR spectra, the peak of 2,2'-(2,2,3,3,4,4,5,5-Octafluorohexane-1,6-diyl) bis (oxirane) at 2930 cm^{-1} came from the -CH- bonding of the saturated carbon atoms in the epoxy groups. It was accompanied by the characteristic peak of epoxy groups at 860 cm^{-1}, which indicated the presence of epoxy groups in the fluoride. The peak at 1350~1200 cm^{-1} was characteristic for the C-F stretching vibration and 900~700 cm^{-1} for the C-F bending vibration. The symmetric and asymmetric -CF_2- stretching had strong absorption in the characteristic peak at 1200-1100 cm^{-1} region. As for the GAP spectra, the strong peak at 2098 cm^{-1} was characteristic for azide groups. The medium strong peak at 3394 cm^{-1} came from hydroxyl groups. The medium strong peaks at 2939 cm^{-1} and 2861 cm^{-1} were attributed to the symmetric and asymmetric methylene stretching vibration. The strong peaks at 1082 cm^{-1} and 1274 cm^{-1} were the stretching vibration peaks of ether bonds, while the one at 933 cm^{-1} was the bending vibration peak of ether bonds. It was worth noticing that the characteristic peaks for epoxy groups at 860 cm^{-1} disappeared. This was indicative that expoxy groups in fluoride were fully

reacted.

Table S2. The values of molar gravitational constant (F).

Group type	$F(J/cm^3)^{1/2}/mol$	Group type	$F(J/cm^3)^{1/2}/mol$
$-CH_3$	450.0	>CH-	60.8
$-CH_2-$	292.0	-CH=	309.0
-O- (ether)	200.0	$-CF_2-$	426.2
-COO-	730.0	=N-	164.4
-OH	525.0	$-N_3$	389.9

Estimation of solubility parameter for G-F-G via the group contribution method:

The solubility parameter (δ) of organics could be used to estimate the solubility of certain organic matter in specific solvent. The group-contribution method[8] could be utilized to approximately calculate the δ values of organic polymers. Therefore, δ was an important index for describing the formation of G-F-G vesicles in this work, which was determined by the square root of the cohesive energy density (CED):

$$\delta = \sqrt{\mathrm{CED}} = [\frac{\Delta H_{vap} - RT}{V_m}]^{1/2} = [\frac{E_m}{V_m}]^{1/2} \tag{1}$$

Where ΔHvap was the heat of vaporization, R was the ideal gas constant, Vm was the molar volume of the solute, and Em was the molar cohesion energy. Em of a liquid was defined as the required energy to break all intermolecular contacts in a mole of liquid. Therefore, it was equal to the difference in the internal energy of vaporization to an ideal gas.

According to the Hansen solubility parameter, CED was originated from the molecular forces including dispersion forces, polar forces, or hydrogen bonding interactions (as in Equation 2), which generally determined the solubility in the mixture.

$$\mathrm{CED} = \delta_d^2 + \delta_p^2 + \delta_h^2 \tag{2}$$

Where δ_d represented the dispersion force component, δ_p represented the polarity force component, and δ_h represented the hydrogen bonding force component.

When two substances were mixed, the heat of mixing depends on the difference in cohesion energy between the solution and the unmixed solute component. G. Scatchard[9] proposed a semi-empirical relationship for the situations where the change in energy of a pair of different molecules (solute vs. solvent) was approximately equal to the geometric mean of the corresponding molecular energy when one of these forces was dominant:

$$\Delta H = \phi_1\phi_2[\left(\frac{E_1}{V_1}\right)^{\frac{1}{2}} - \left(\frac{E_2}{V_2}\right)^{\frac{1}{2}}]^2 \tag{3}$$

Where ΔH was heat of mixing of mixture, ϕ_1 and ϕ_2 were volume fraction of different components, where E and V were molar cohesive energies and molar volumes, respectively.

The Scatchard equation (3) was equivalent to the statement: when n_1 mol of liquid 1 with molar volume V_1 and cohesive energy E_1 was mixed with n_2 mol of liquid 2 with molar volume V_2 and cohesive energy E_2, it had cohesive energy E:

$$E^{\frac{1}{2}}(n_1V_1 + n_2V_2)^{1/2} = n_1(E_1n_1)^{1/2} + n_2(E_2n_2)^{1/2} \tag{4}$$

Herein, $(EV)^{1/2}$ had additivity, reasonable to add on the basis of the atoms and composition of the compounds. It was possible to find a group of additive constants for the groups in an organic molecule and thus $(EV)^{1/2}$ could be calculated. P. A. Small[10] defines $(EV)^{1/2}$ as the molar attraction constant, denoted by the symbol F. Therefore, δ could be expressed as follows:

$$\delta = \sqrt{\frac{E_m}{V_m}} = \frac{\sqrt{E_mV_m}}{V_m} = \sum_i \frac{n_iF_i}{V_m} \tag{5}$$

Where, F_i was the molar gravitational constant of one certain functional group, n_i was the number of one certain functional group.

The molar gravitational constant values (F) had been revised according to some researchers[11]. The revised F values could be obtained by referring to **Table S3**. In order to facilitate calculation with readily available data, the molar volume was expressed in terms of density (ρ) divided by molar mass (M)：

$$\delta = \frac{\rho}{M}\sum_i n_iF_i \tag{6}$$

M and ρ of G-F-G copolymer were calculated to be 1139 g/mol, and 0.85 g/cm^3. The solubility parameters of G-F-G are obtained by substituting the numerical values into **Equation 6**:

$$\delta_{G-F-G} = 11.1\ J^{\frac{1}{2}} \cdot cm^{-\frac{3}{2}} = 22.6\ cal^{\frac{1}{2}} \cdot cm^{-\frac{3}{2}}$$

The solubility parameter (δ) calculated by this method could be an estimate for qualitative analysis. In fact, the formation of G-F-G vesicles were not only related to solubility parameters, but also closely related to polarity, functional groups， concentration, stirring mode/rate and other parameters[12]. The solubility parameters (at room temperature) of AE, THF and DMF used in this paper were 12.9, 9.2 and 12.1 cal$^{1/2}$cm$^{-3/2}$, respectively. The magnitude of polarity could be expressed by the permittivity[13]. Their permittivities (at room temperature) were 24.3, 7.58, and 36.7 F/m, respectively. The greater the permittivity of the solvent was, the greater the polarity of the solvent should be. For the polar polymer of G-F-G, the polarity of the solvent had a great influence on solubility. Compared with AE and THF, DMF with the relatively high polarity had better solubility for G-F-G. This was in accordance with our experimental results that G-F-G could be well dissolved in DMF while G-F-G particles remained in AE or THF. As the solubility parameter of G-F-G was larger than that of DMF, G-F-G molecules tended to aggregate and exist in the form of self-assembled vesicles rather than fully dissolved polymer coils. The estimation of solubility parameters confirmed the proposed mechanisms for the formation of G-F-G vesicles, which could help to select potentially suitable solvents for achieving them.

Table S3. The organic coating mass fraction in Al-based fuels determined by EDTA titration.

Sample	Al@G-F-G(DMF)	Al@G-F-G(THF)	Al@G-F-G(AE)	Al/F/GAP
Coating mass fraction (%)	2.27%	5.84%	5.32%	13.7%

Table S4. The specific surface areas of the Al powder samples.

Sample	Specific area (m^2/g)	Specific surface area growth rate (%)
Raw Al	5.55	0.0
Al@G-F-G (DMF)	9.23	66.3
Al/F/GAP	3.56	-35.9

2 Supplementary Figures

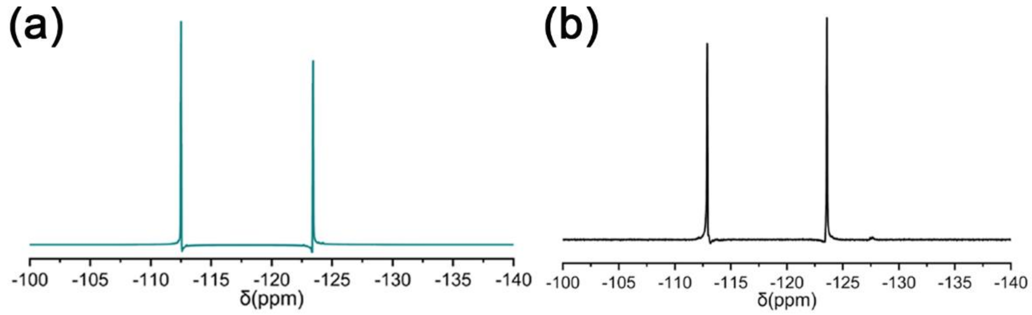

Figure S1. F-NMR spectra of fluoride (a) and G-F-G copolymer (b).

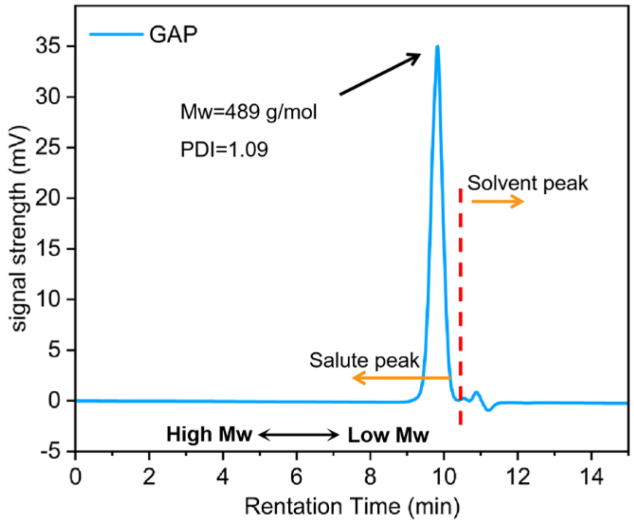

Figure S2. The GPC curve of GAP.

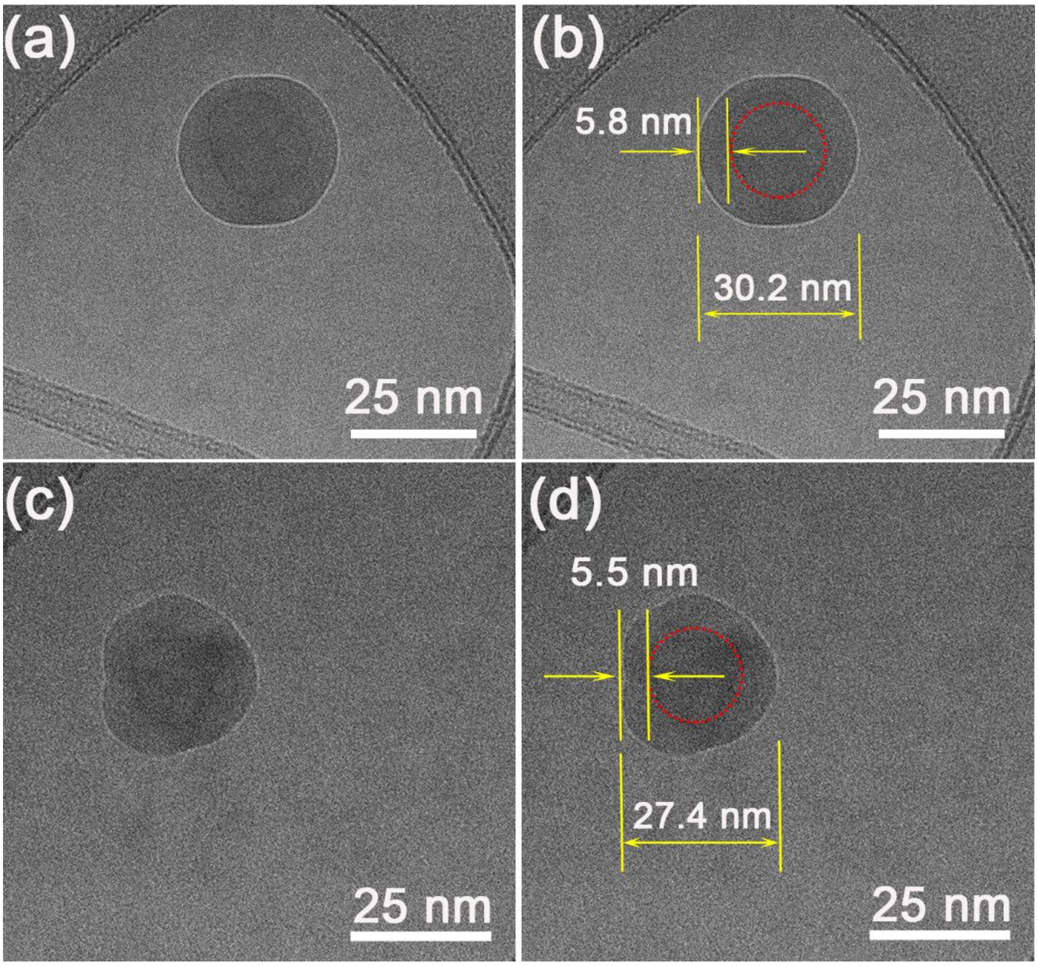

Figure S3. The Cryo-TEM images of water-treated vesicles (a, c) and the corresponding dimensions (b, d). The corresponding shell-core structure for vesicles could be observed and the size was in accordance with our previously obtained results.

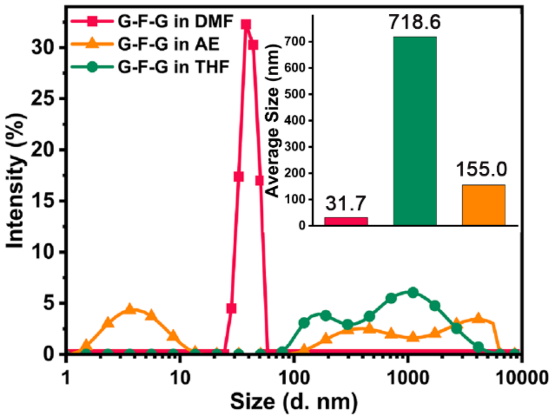

Figure S4. The size distribution of G-F-G vesicles in DMF, THF and AE.

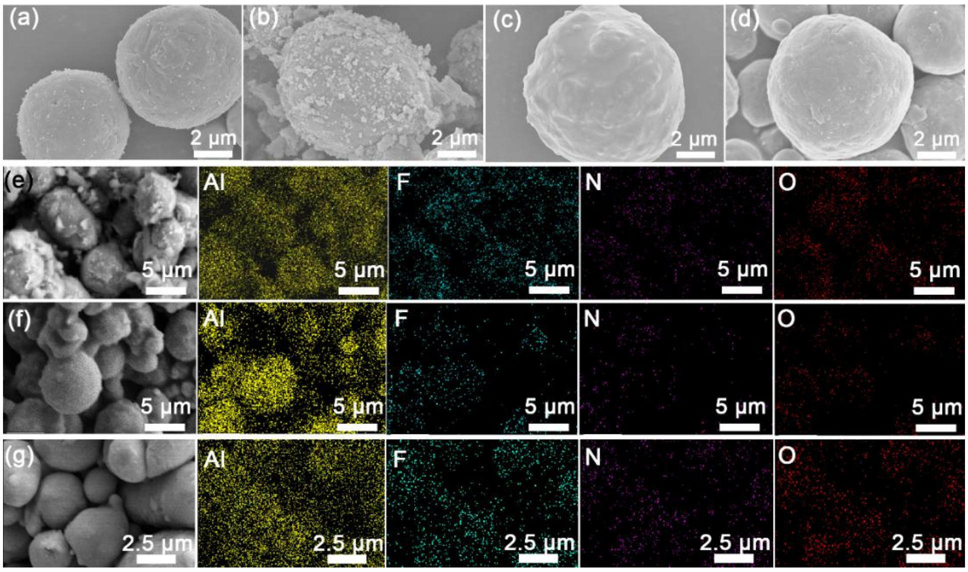

Figure S5. The SEM images of raw Al (a), Al@G-F-G(THF) (b), Al@G-F-G(AE) (c) and Al/F/GAP (d); element mapping of Al@G-F-G(THF) (e), Al@G-F-G(AE) (f) and Al/F/GAP (g).

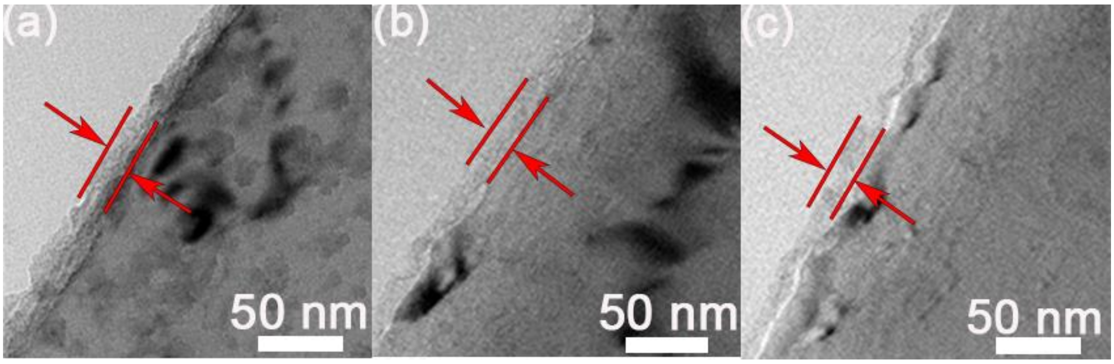

Figure S6. Multiple cross-sectional TEM images of Al@G-F-G(DMF) samples.

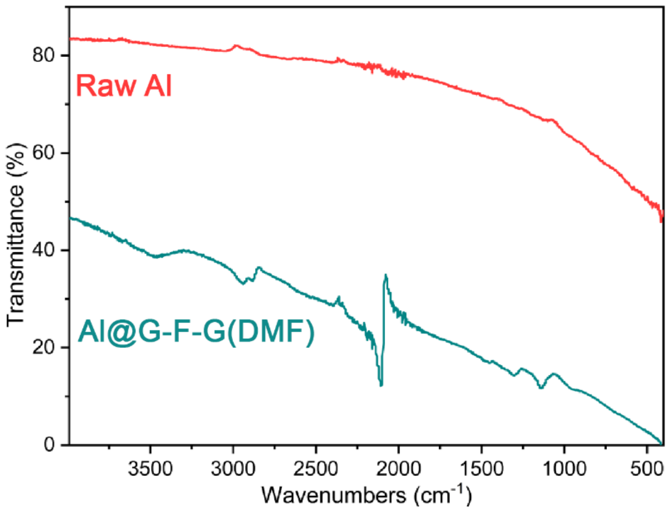

Figure S7. FTIR curves of raw Al and Al@G-F-G(DMF).

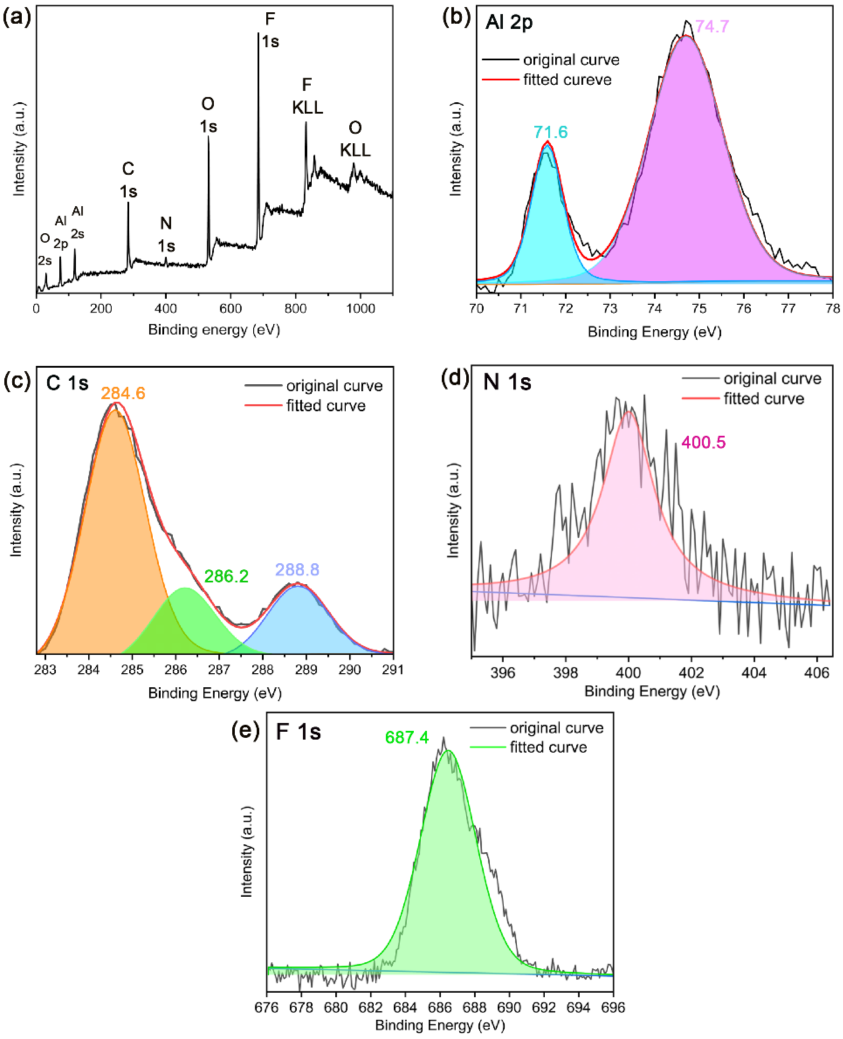

Figure S8. XPS spectra of total (a), Al 2p (b), C 1s (c), N 1s (d) and F 1s (e) of Al@G-F-G(DMF).

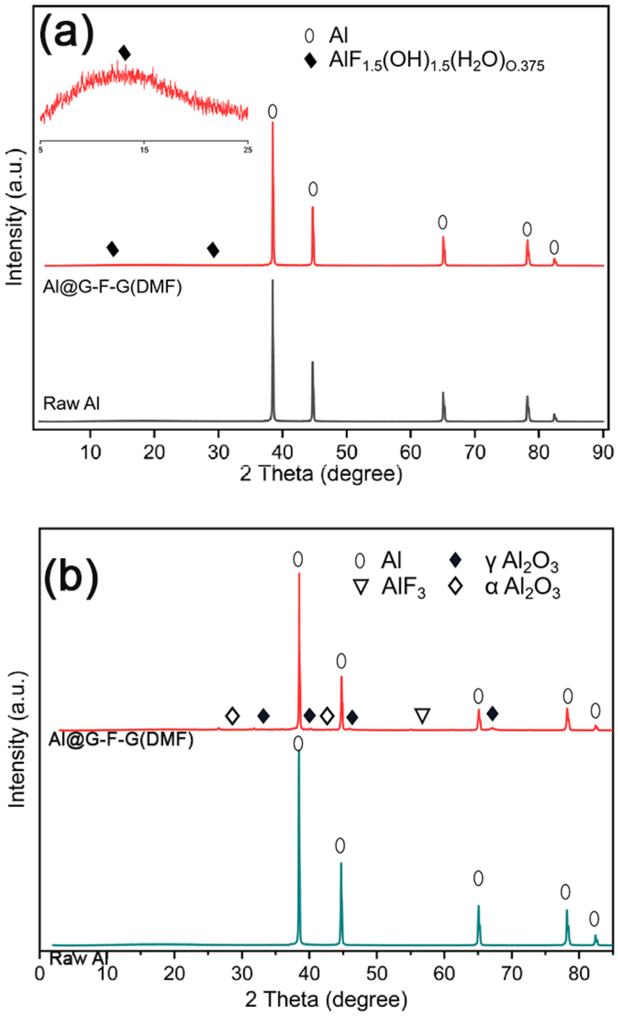

Figure S9. XRD curves of raw Al, Al@G-F-G(DMF) (a) and corresponding XRD curves of combustion products (b).

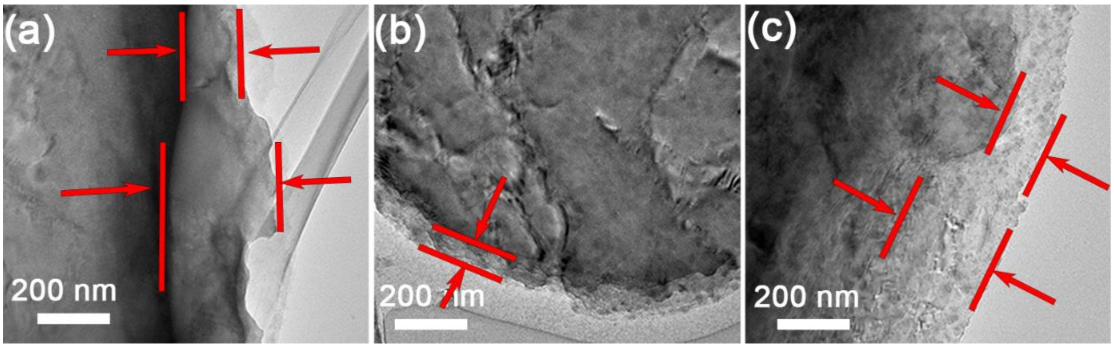

Figure S10. Cross-sectional TEM and HRTEM images of Al@G-F-G(THF) (a), Al@G-F-G(AE) (b) and Al/F/GAP (c).

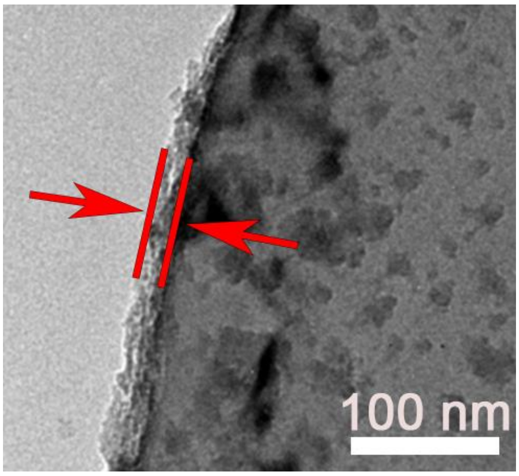

Figure S11. The TEM image of the cross-sectional area for Al@G-F-G(DMF) sample prepared with higher G-F-G concentration (doubled comparing to the original one).

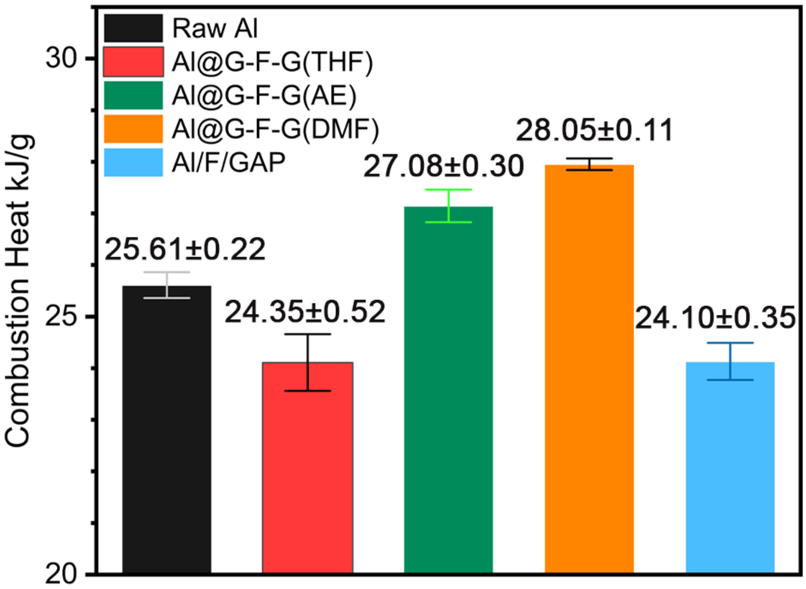

Figure S12. The statistic data with error bars for the combustion heat of the tested samples.

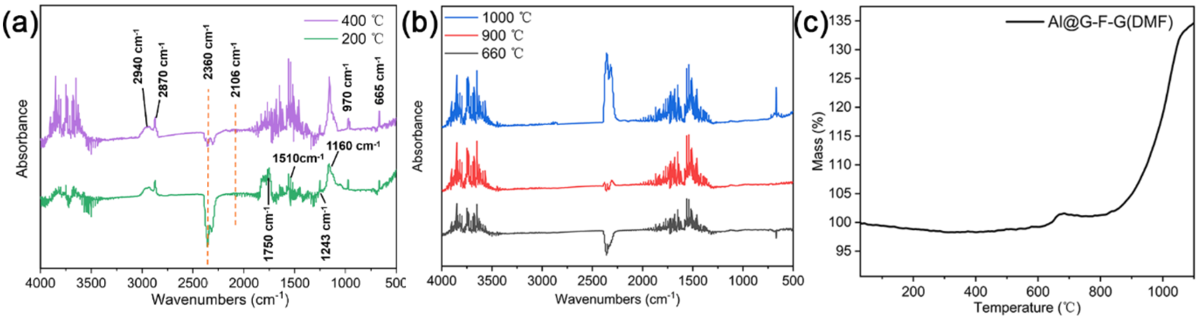

Figure S13. TG/FTIR curves of Al@G-F-G(DMF).

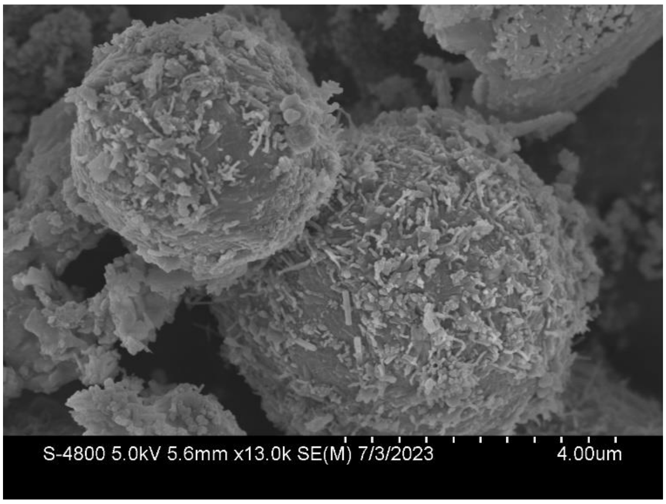

Figure S14. Dense coating of AlF_3.

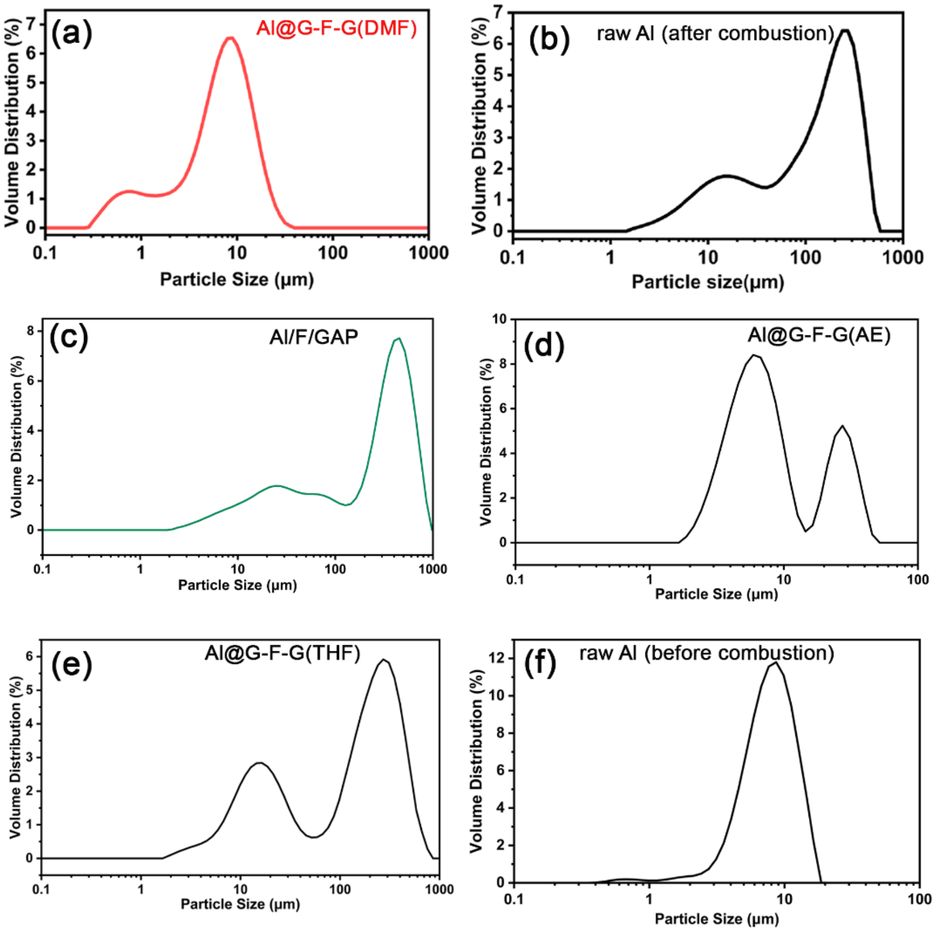

Figure S15. Volume distribution for the combustion product of Al@G-F-G(DMF) (a), raw Al after combustion (b), Al/F/GAP (c), Al@G-F-G(AE) (d), Al@G-F-G(THF) (e) and raw Al before combustion (f)

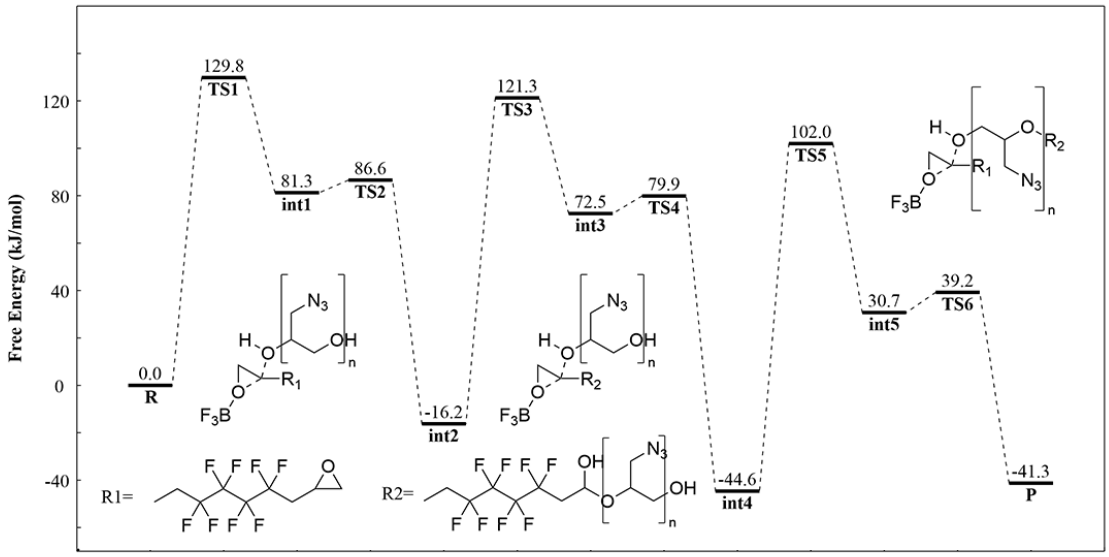

Figure S16. Reaction energy profiles for G-F-G chemistry.

3 Simulation calculation of G-F-G synthetic chemistry

According to **Figure S16**, the structure of reactants, products and reaction intermediates were optimized under the framework of density functional theory (DFT) with m062x functional[14] and 6-31G (d, p) basis set. The transition state was also searched by using the same functional and basis set. The vibrational frequency analysis was carried out for the optimized structure with the same calculation method. In order to describe the solvation effect, the SMD (Solvation Model Based on Density)[15] implicit solvent model was used in all calculations. The thermodynamic correction terms and Gibbs free energy of their structures at 333.15 K (55 °C) were then obtained by using Shermo program[16]. In order to obtain the electron energy with higher accuracy, a single point calculation for these optimized structures with M06-2x functional and def2-TZVP basis set were performed. Finally, the single point energy was added to the free energy correction calculated before to obtain the Gibbs free energy. All these DFT calculations were performed using Gaussian 16 program suite[17]. The calculation includes six transition states and five intermediate states. According to simulation calculation, the activation energy of GAP and fluoride reaction was 146 kJ/mol, and the activation energy of GAP and G-F-G reaction was 137 kJ/mol. The

deviation was 9 kJ/mol. According to the theory of chemical reaction kinetics, the difference in the chemical reactivity for two routines was $e^{(\mathrm{delta\ Ea/RT})} = 38$ times. The free energy of GAP reaction with fluoride was a negative value of -44.60 kJ/mol. The free energy of fluoride and G-F-G reaction was positive value of 3.34 kJ/mol. According to the calculation results, it was difficult for the side reaction between fluoride and G-F-G to occur in our work.

Reference

[1] S. Y. Wu, X. M. Li, Z. Ge, Y. J. Luo, *Polymers* **2023**, *15* (6), https://doi.org/10.3390/polym15061538.

[2] K. K. S. Lau, J. Bico, K. B. K. Teo, M. Chhowalla, G. A. J. Amaratunga, W. I. Milne, G. H. McKinley, K. K. Gleason, *Nano Letters* **2003**, *3* (12), 1701, https://doi.org/10.1021/nl034704t.

[3] B. C. Smith, *Spectroscopy* **2022**, *37* (3), 17.

[4] H. F. Shurvell, D. W. Hyslop, *Journal of Chemical Physics* **1970**, *52* (2), 881, https://doi.org/10.1063/1.1673068.

[5] a) L. C. Zhang, X. Su, S. Wang, X. D. Li, M. S. Zou, *Chemical Engineering Journal* **2022**, *450*, https://doi.org/10.1016/j.cej.2022.137118; b) A. C. Jones, M. B. Raschke, *Nano Letters* **2012**, *12* (3), 1475, https://doi.org/10.1021/nl204201g.

[6] D. W. Kim, K. T. Kim, T. S. Min, K. J. Kim, S. H. Kim, *Scientific Reports* **2017**, *7*, https://doi.org/10.1038/s41598-017-04758-7.

[7] A. Bagri, C. Mattevi, M. Acik, Y. J. Chabal, M. Chhowalla, V. B. Shenoy, *Nature Chemistry* **2010**, *2* (7), 581, https://doi.org/10.1038/nchem.686.

[8] V. van Speybroeck, R. Gani, R. J. Meier, *Chemical Society Reviews* **2010**, *39* (5), 1764, https://doi.org/10.1039/b809850f.

[9] G. Scatchard, *Annals of the New York Academy of Sciences* **1949**, *51* (4), 660, https://doi.org/10.1111/j.1749-6632.1949.tb27297.x.

[10] P. A. Small, *Journal of Applied Chemistry* **1953**, *3* (2), 71.

[11] Z. Li, Z. Ling, X. Ying, G. Liu, **2002**, *53*, 1288.

[12] Y. Lu, E. Zhang, J. Yang, Z. Cao, *Nano Res* **2018**, *11* (10), 4985, https://doi.org/10.1007/s12274-018-2152-3.

[13] X. D. Hong, Z. Y. Xu, Z. P. Lv, Z. Lin, M. Ahmadi, L. F. Cui, V. Liljeström, V. Dudko, J. L. Sheng, X. Q. Cui, A. P. Tsapenko, J. Breu, Z. P. Sun, Q. Zhang, E. Kauppinen, B. Peng, O. Ikkala, *Advanced Science* **2023**, https://doi.org/10.1002/advs.202305099.

[14] Y. Zhao, D. G. Truhlar, *Theoretical Chemistry Accounts* **2008**, *120* (1-3), 215, https://doi.org/10.1007/s00214-007-0310-x.

[15] A. V. Marenich, C. J. Cramer, D. G. Truhlar, *The Journal of Physical Chemistry B* **2009**, *113* (18), 6378, https://doi.org/10.1021/jp810292n.

[16] T. Lu, Q. Chen, *Computational and Theoretical Chemistry* **2021**, *1200*, 113249, https://doi.org/https://doi.org/10.1016/j.comptc.2021.113249.

[17] M. J. Frisch, G. W. Trucks, H. B. Schlegel, G. E. Scuseria, M. A. Robb, J. R. Cheeseman, G.

Scalmani, V. Barone, G. A. Petersson, H. Nakatsuji, X. Li, M. Caricato, A. V. Marenich, J. Bloino, B. G. Janesko, R. Gomperts, B. Mennucci, H. P. Hratchian, J. V. Ortiz, A. F. Izmaylov, J. L. Sonnenberg, Williams, F. Ding, F. Lipparini, F. Egidi, J. Goings, B. Peng, A. Petrone, T. Henderson, D. Ranasinghe, V. G. Zakrzewski, J. Gao, N. Rega, G. Zheng, W. Liang, M. Hada, M. Ehara, K. Toyota, R. Fukuda, J. Hasegawa, M. Ishida, T. Nakajima, Y. Honda, O. Kitao, H. Nakai, T. Vreven, K. Throssell, J. A. Montgomery Jr., J. E. Peralta, F. Ogliaro, M. J. Bearpark, J. J. Heyd, E. N. Brothers, K. N. Kudin, V. N. Staroverov, T. A. Keith, R. Kobayashi, J. Normand, K. Raghavachari, A. P. Rendell, J. C. Burant, S. S. Iyengar, J. Tomasi, M. Cossi, J. M. Millam, M. Klene, C. Adamo, R. Cammi, J. W. Ochterski, R. L. Martin, K. Morokuma, O. Farkas, J. B. Foresman, D. J. Fox, Gaussian 16 Rev. C.01. Wallingford, CT, **2016**.
